# ‘I Would Feel Failed By the Medical Community Entirely’: Transgender Americans' Calls for Advocacy From Health Professionals in a Care‐Restricted World

**DOI:** 10.1111/hex.70716

**Published:** 2026-06-12

**Authors:** Kay Wallace, Jayne Reynolds, Jesse Krikorian, Gaines Blasdel, Mary Byrnes, Daphna Stroumsa

**Affiliations:** ^1^ University of Michigan Medical School Ann Arbor MI USA; ^2^ Department of Obstetrics and Gynecology University of Michigan Ann Arbor MI USA; ^3^ Department of Family Medicine University of Michigan Ann Arbor MI USA; ^4^ Department of Surgery University of Michigan Ann Arbor MI USA

**Keywords:** gender‐affirming care, patient advocacy, transgender health

## Abstract

**Background:**

Gender‐affirming care (GAC) improves the mental health, quality of life, and social functioning of gender‐diverse patients. In spite of this, GAC in the United States (as elsewhere) has become a major focus of debate, with several federal policy changes enacted through Executive Orders and legislation creating major restrictions on transgender (trans) Americans' ability to obtain the care they need.

**Objective:**

We aimed to understand how trans Americans are responding to the anticipated changes in their access to GAC and to provide healthcare professionals with strategies to support this population in an environment of restricted access.

**Design:**

We surveyed trans people in the United States to elicit their concerns and planning considerations. We used descriptive statistics to characterise coping strategies and content analysis to describe support strategies that the trans community requested from the healthcare community.

**Setting and Participants:**

We recruited participants from December 2024 through March 2025 using snowball sampling and direct outreach to LGBTQ+ organisations. Participants self‐identified as trans and were 18 or older.

**Results:**

Of 157 respondents, 96.2% (*n* = 151) expressed concern about access to GAC. We describe five strategies for healthcare support: mental health support/suicide prevention, financial support, connections with community organisations/mutual aid, GAC education, and advocacy.

**Discussion:**

The current politicisation of transgender healthcare is affecting trans people, with the vast majority concerned about barriers that will make this care unattainable. It is essential for clinicians to understand the responses to these policy changes from the trans community and to act on their calls for support.

**Conclusions:**

Healthcare providers stand at a crossroads with the trans community. If clinicians fail to stand up for the rights of trans patients, we risk irrevocably harming the medical trust and long‐term health of the trans community.

**Patient or Public Contribution:**

Patients and people with lived experience were heavily involved in the design, conduct, and analysis of this study. The authorship group included a range of gender identities and expressions, including people accessing GAC as patients and clinicians providing GAC. Analysis was guided by participant feedback and iterative discussion among the authorship group.

## 1 Introduction

Gender‐affirming care (GAC) improves the mental health, quality of life, and social functioning of transgender and nonbinary (trans) patients [1, 2, 3] and is supported by all relevant major U.S. medical associations [4, 5, 6, 7, 8]. Despite this fact, GAC has become an increasing target of political discourse in recent years, and in particular, during the second Trump presidency. On January 20, 2025, Executive Order (EO) 14168 was published, which restricted the federal government's definition of gender to two sexes [9]. This was quickly followed by EO 14187 on January 28, 2025, which described GAC for patients under age 19 as ‘chemical and surgical mutilation’ and officially blocked access to and federal funding for GAC for this population, affecting both children and legal adults of age 18 [10]. On June 18th, 2025, the Supreme Court decided in the case of *United States versus Skrmetti* that state bans on GAC for youth are not subject to heightened scrutiny, thus enabling continued legislation banning access to such care [11].

The outsized political and legislative focus on a group that makes up approximately 1% of the U.S. population [12] comes with major consequences for the healthcare needs of trans Americans, a group already marginalised and facing healthcare disparities. Trans people experience higher rates of depression, anxiety, suicidal ideation, and other mental health conditions [13]; are more likely to report poor physical health, experience higher rates of major health conditions such as asthma and cardiovascular disease, and face disproportionate risk of HIV and substance use disorder compared to cisgender people [14]; and report high rates of discrimination in healthcare settings [15]. The 2022 U.S. Trans Survey reported that 47% of trans participants had at least one negative interaction with a healthcare provider, 24% avoided healthcare in the past year due to fear of being mistreated as a trans person, and 2% faced physical or sexual violence when seeking medical care [16]. Combined with financial and insurance status factors, these experiences lead trans patients to delay seeking and accessing healthcare services [17]. The widening legislative and political restrictions on GAC, as well as on trans rights more broadly, threaten to accelerate these inequities and their subsequent negative health effects on the trans community.

In light of the increasing negative impact of enacted policies on trans health equity, healthcare providers have a central role in understanding the needs of and advocating for their trans patients. Access to good health and compassionate care is among the most fundamental of the human rights violations currently being inflicted on the trans community. In an environment in which a net of discrimination is tightening legislatively, politically, socially, and economically, healthcare providers are among the few professions of influence able to stand up for trans rights and to make meaningful, positive change for their trans patients. It is therefore not only necessary, but ethically imperative, to understand and respond to the changing healthcare needs of trans patients, and to appreciate and respond to their requests for advocacy from their providers. We therefore aimed to understand how trans Americans are responding to the anticipated changes to their ability to access healthcare and to provide healthcare professionals with the tools and information necessary to support the trans community in reducing harm and increasing well‐being in this environment.

## 2 Materials and Methods

### 2.1 Study Design

We designed a survey study with both closed‐ and open‐ended responses to understand and describe how trans Americans anticipated healthcare policy changes and to allow for a holistic understanding of participant concerns. The 37‐item, approximately 10‐min electronic survey included questions on demographics, current and past medical gender‐affirming treatment, concerns regarding transgender healthcare, and participants' responses to those concerns *(Appendix)*. Our institutional review board deemed this study exempt (HUM00265503). The opening of the survey described the purpose of the study, risks and benefits of participation (including an acknowledgement that the survey's contents may be potentially distressing, as well as the option to decline to answer any question), and a description of confidentiality procedures. Survey continuation implied consent to participate. Previous studies have demonstrated that survey questions discussing restrictions on GAC can be distressing for participants and can lead to disclosure of mental health struggles and even suicidality [18]. Therefore, the closing of the survey included text urging participants to reach out if they were facing mental health challenges or were feeling suicidal; contact information for the national suicide and crisis hotline and the Trans Lifeline were also provided.

### 2.2 Sampling and Recruitment

We recruited participants from December 2024 through March 2025 using snowball sampling and direct outreach to organisations that serve LGBTQ+ populations. We chose this survey methodology to extend recruitment reach, as the trans community can be considered a hidden population. We contacted participants and organisations via email and/or phone call with a description of the study, an email address for questions, the IRB approval number, compensation details, and a link to the survey. We made a second attempt at contact at 2 weeks if the first attempt was unsuccessful. Inclusion criteria were trans self‐identification and age ≥ 18. In January 2025, initial demographics revealed a disproportionately White study population. In order to improve the racial and ethnic diversity of our sample, we adjusted recruitment toward organisations serving trans communities of colour and amended our study description to include purposive sampling to ensure a diverse and representative sample.

### 2.3 Data Collection

Survey distribution began shortly after the U.S. general elections and continued through the period following the issuance of the EOs (December 2024‐March 2025). Qualtrics reCAPTCHA scores were enabled to aid in detection of fraudulent responses. Responses were excluded if reCAPTCHA scores indicated bot involvement (score < 0.5 on 0‐1 scale), or if the participant did not provide an address and none of the open‐ended questions were completed. Participants who completed the survey and provided an address received a $50 gift card via mail as compensation.

### 2.4 Analysis

Analysis occurred between April and July 2025. Closed‐ended survey questions were collected and analysed in Excel, which we used to generate descriptive statistics. Quantitative analysis enabled us to characterise types of strategies that participants selected in response to an increasingly restrictive GAC environment. We approached open‐ended survey questions through inductive content analysis as described by Elo and Kyngäs [19] in order to synthesise support requests from the trans community to healthcare providers. Throughout the process of data collection and analysis, the research team extensively and iteratively discussed the ethics of the project and its framing, taking into account participants' requests to centre the study's focus on healthcare provider advocacy. Therefore, responses to the survey item ‘If accessing transition‐related care becomes (more) difficult or not possible, what kind of support would be valuable to you ‐ from healthcare professionals, community organisations, or other sources?' were used as the unit of analysis. We analysed data in three phases: preparation, organising, and abstraction. In the preparation phase, all responses were read multiple times to achieve immersion in the data. In the organisation phase, open coding was performed by identifying and labelling meaningful segments of responses. Codes were compiled in a coding sheet and grouped together into subcategories based on content similarity. In the abstraction phase, subcategories with similar content were grouped into main categories generated by the patterns and relationships identified in the data. We selected representative quotations to illustrate each category, ensuring transparency and depth in reporting.

### 2.5 Reflexivity

The authorship group included people accessing GAC as patients, clinicians providing GAC, medical students with clinical interest in GAC, and researchers with experience in the field of transgender health. A range of gender identities and expressions were represented in the group. Potential bias was minimised through iterative discussions within the study team.

## 3 Results

### 3.1 Demographics

After excluding 87 responses that were flagged as likely fraudulent or duplicate responses per Qualtrics' ReCAPTCHA score and by manual review, our final sample was 157 unique respondents. A large majority (*n* = 127, 80.1%) of participants were assigned female at birth (AFAB), 17.8% (*n* = 28) were assigned male at birth (AMAB), and 1.3% (*n* = 2) were intersex (Table 1). We used open‐ended questions to elicit gender identity, resulting in several overlapping gender categories: 101 (64.3%) included a broadly masculine term (e.g., male, man, trans male, trans masc, trans man, M, FTM); 24 (15.3%) included a broadly feminine term (e.g., female, woman, MTF, trans woman, trans femme/female/feminine); and 49 (31.2%) used non‐binary terms (e.g., two spirit, agender, non‐binary, queer or genderqueer). At the time they completed the survey, 132 participants (84.1%) were using gender‐affirming hormones and 25 (15.9%) were not.

**Table 1:** Participant demographics.

Characteristic	*N* (%) or Mean ± SD
Age, y	38 ± 11.4
Sex Assigned at birth (*N* = 157)	
Female	127 (80.9)
Male	28 (17.8)
Intersex	2 (1.3)
Gender identity^a^ (N = 157)	
Masculine	101 (64.3)
Feminine	24 (15.3)
Non‐binary	49 (31.2)
Race (N = 142)	
American Indian or Alaska Native	1 (0.7)
Asian, Hawaiian, or Pacific Islander	2 (1.4)
Black or African‐American	6 (4.2)
Middle Eastern or Mediterranean	1 (0.7)
White	127 (89.4)
Other or prefer to not disclose	5 (3.5)
Hispanic or Latinx (N = 157)	
Yes	14 (8.9)
No	143 (91.1)
Location (N = 157)	
Urban	73 (46.5)
Suburban	51 (32.5)
Rural	33 (21.0)
Region (N = 157)	
Northeast	42 (26.8)
Midwest	53 (33.8)
West	30 (19.1)
South	30 (19.1)
Outside the continental U.S.	2 (1.3)
Level of Education (N = 157)	
< High school	5 (3.2)
High school graduate	9 (5.7)
Some college	26 (16.6)
Professional degree	14 (8.9)
4‐year or college degree	42 (26.8)
Advanced degree	61 (38.9)
Employment Status (N = 155)	
Unemployed	18 (11.6)
Employed part time	18 (11.6)
Employed full time	89 (57.4)
Self‐employed	13 (8.4)
Full‐time student	6 (3.9)
Other	11 (7.1)
Household Income (N = 131)	
< = $10,000	8 (6.1)
%10,000‐$19,999	9 (6.9)
$20,000‐$39,999	20 (15.3)
$40,000‐$59,999	14 (10.7)
$60,000‐$79,999	19 (14.5)
$80,000‐$99,999	14 (10.7)
$100,000‐$149,999	26 (19.8)
≥ = $150,000	21 (16.0)
Insurance Status (N = 157)	
Not insured	6 (3.8)
Medicaid, Medicare, Indian Health Service, or other public insurance	34 (21.7)
VA	0 (0)
Private insurance through an employer	88 (56.1)
Private insurance through the marketplace	17 (10.8)
Other	12 (7.6)
Currently on Gender‐affirming Hormone Therapy (N = 157)	
Yes	132 (84.1)
No	25 (15.9)
Coping Strategies^b^ (N = 157)	
Community‐based support	127 (80.9)
Geographical relocation	48 (30.6)
Changes to legal documentation	64 (40.8)
Modifying gender expression	42 (26.8)
De/re‐transitioning	2 (1.3)

^a^ Open‐ended question; some participants included multiple identity terms or terms that fit into more than one category.

^b^ Participants could choose more than one answer.

Participants' ages ranged from 19 to 76 years, with a mean age of 38.0 ± 11.4 years. Respondents came from 30 states, as well as the District of Columbia. Two respondents were U.S. citizens living abroad. Most respondents identified as White (127/142, 80.9%) and non‐Hispanic (143/157, 91.1%).

### 3.2 Access Concerns

Participants reported extremely high levels of concern regarding access to GAC: 99.4% (156/157) were somewhat or very worried about *others'* access to hormones over the next 4 years, 85.3% (134/157) were somewhat or very worried about *their own* access to hormones over the next 4 years, and 89.8% (141/157) stated that their feelings about their own ability to access hormones had changed following the 2024 elections. Of the 75 participants who were interested in gender‐affirming surgery, 88% (*n* = 66) expressed concern about future access to surgery. The lower response rate for this item was likely due to the fact that many participants indicated they were not interested in new or further gender‐affirming surgery (*n* = 63) or that they were currently unsure of plans for new or further surgery (*n* = 13).

### 3.3 Support From Healthcare Providers

In their responses to open‐ended questions, participants offered a plethora of strategies for providers to support trans community members. Inductive qualitative analysis of support requests yielded five broad categories: mental health support and suicide prevention, financial support, connections with community organisations, patient education, and advocacy.

#### 3.3.1

Twenty‐four (15.2%) participants described mental health and emotional support as valuable strategies their healthcare providers could undertake should access to care become more restricted. These requests included general mental health check‐ins, referrals to therapists or support groups, and frank discussions with healthcare providers about the effects of care restriction on mental health. ‘My doctor discussed these possible changes with me and also made space for my feelings around this stuff,’ said one participant, ‘It was something I didn't know I needed, just to have the pain and fear acknowledged.’ ‘[We need] general mental health check‐ins,’ said another, ‘Every trans person has to be feeling the heat right now.’ As participants discussed, acknowledgement of the mental load of increased political and legislative scrutiny of trans patients demonstrates efforts to understand of the trans experience and can help strengthen therapeutic alliance, while serving as a key leverage point of intervention for trans patients requiring increased mental health support.

Further, eleven (7.0%) participants expressed concern about suicidality, both personal and community‐wide—a concern that is supported by extensive data showing a causal relationship between GAC bans and suicidality of trans youth in the United States [20]. Two respondents named an intent to die by suicide if they were not able to access GAC, while three others described suicidal thoughts in the past that resolved around GAC, or referred to the medication as ‘life‐saving’ or ‘life or death.’ An additional seven respondents expressed fear that other trans people would die by suicide as a result of being unable to access care. Increasing prevalence of suicidal ideation in this population requires aggressive prevention strategies from clinicians, including screening, counselling, coordination of psychiatric care, and raising awareness among colleagues of the accelerating mental health needs of trans patients in a restrictive care environment.

#### 3.3.2 Financial Support

Seventeen (10.8%) participants described financial support from providers as a valuable support strategy in an environment of restricted access to care. Participants particularly worried that restrictions on insurance coverage for GAC would significantly increase their healthcare costs. Sliding scale models, pro bono work, crowdfunding, and support for navigating changes to insurance coverage were all mentioned as actions to address this concern. Participants stressed the need for support for trans patients with multiple marginalised identities: ‘[We need] more funding for financial assistance for QTBIPOC and marginalised peoples. It's only gonna be harder and [I] already have friends looking for 3rd jobs (myself included).’ Financial support was also mentioned as a prerequisite for access to other suggested support strategies, including mental health. ‘Many of us, myself included, cannot afford [mental health services] even with (and sometimes because of) insurance,’ one participant explained, ‘Providers reducing cost, opening up sliding scale systems, etc., for these services will save lives.’ As participants discussed, finding ways to decrease the financial burden of care is key to practically supporting the well‐being of trans patients.

#### 3.3.3 Connections With Community Organisations and Mutual Aid

Twenty‐six (16.6%) participants requested assistance in connecting with community organisations and mutual aid. ‘I would like doctors to partner with mutual aid organisations more,’ said one participant, ‘[We need] a deeper partnership from all kinds… Partner directly with trans support groups, and create new kinds of community organisations to address human needs as they arise.’ Participants discussed resource directories, knowledge of local trans social and political groups, connections to legal services, formation of networks of providers who are continuing to provide GAC, and lists of trans‐affirming medical professionals; for example, one participant expressed the need for ‘actual networks of professionals who provide gender‐affirming care instead of. net type websites with out‐of‐date lists of potentially trans‐friendly providers.’ Other support strategies included having hotline numbers readily available, referrals to non‐medical GAC resources, knowledge of university or employer resources (if available), and connections to mutual aid groups.

#### 3.3.4 Education on the Current Landscape of and Safe Strategies for GAC

Twenty‐three (14.6%) participants cited education on the current landscape of and safe strategies for GAC as a key way for healthcare providers to support trans patients in an environment of restricted access to care. Many of these requests centred on honest, practical discussions of how care might change with legislation or frank discussions about hormone needs after surgery. ‘One type of support that has been particularly valuable to me recently is consideration of changes in access to healthcare when planning long‐term gender‐affirming care,’ explained one participant,

For example, I discussed the possibility of an orchiectomy with my doctor in the past, and ultimately decided against it, partially because of their advice that it could lead to additional health risks if I had to discontinue hormone replacement for some reason and had no endogenous sex hormone production.

These discussions included requests for up‐to‐date information on post‐surgical care as well. As one participant stated, ‘[We need] information about what surgical care is still available. If my erectile implant has an issue, would care for that issue still be covered? [Would] replacement of the device?’ Participants also frequently stressed the need for information on safe sources for continuation of GAC in a restrictive environment, non‐prescription ways to maintain desired physical attributes, and the process of seeking care in other countries if necessary. Participants emphasised the need for ‘clear, relevant, up‐to‐date information about accessing transition related care on both telehealth and local in‐person basis,’ as well as ‘detailed, specific information and resources on how to obtain hormones if hormone access for trans people becomes restricted on a federal level.’ Finally, up‐to‐date information on changes to insurance coverage, counselling from trans‐competent professionals, and ways to manage physical changes if access to GAC were completely lost were all discussed as valuable strategies healthcare professionals could use to support the trans community at this time.

#### 3.3.5 Advocacy

Nineteen (12.1%) participants highlighted advocacy as an important source of support from healthcare providers. ‘Honestly, advocacy from medical professionals would be really important,’ described one participant, ‘I think there is sometimes unwillingness to step into ‘political’ issues, but trans people and the people who provide them care need to push for protections the way the right is pushing restrictions.’ Participants requested direct action from their providers, including lobbying, legislative advocacy, and participating in organising efforts led by the trans community. ‘I also just think showing up and being willing to throw down in defence of trans rights through protest, being loud, and encoding protections will be big,’ explained one participant, ‘The biggest thing we need right now are people willing to fight the legal fight.’ Public allyship was another important aspect of advocacy requests from participants, including public correction of misinformation, education of peers, and openly discussing the essential and life‐saving nature of GAC. ‘The most important thing for me would be for them to become vocal and visible advocates for the need for providing medical care to trans people,’ one participant said. ‘[We need] public education,’ described another, ‘There is so much misinformation. The misinformation is being weaponized against us… It's a confusing world in which to access accurate information.’ Healthcare providers are in a unique position to translate both scientific, medical, and legal information into understandable language for both trans patients and the public at large. Using that voice to educate patients, colleagues, lawmakers, and the public is just one of many ways way that healthcare providers can advocate on behalf of trans people. As one participant summarised: ‘From community groups to medical orgs, all those that want to paste rainbows in June, I'd hope that they all step up to show their support in the other 11 months.’

## 4 Discussion

The current politicisation of transgender healthcare is affecting trans people, with the vast majority concerned about barriers that will make this care unattainable. It is essential for clinicians to understand trans people's responses to the social environment and ongoing policy changes, and to act on their calls for support.

The role of physicians as advocates for marginalised populations is well‐established. Physicians have led and supported efforts for a variety of marginalised groups, including playing a pivotal role in the establishment of Federally Qualified Health Centers and expansion of health insurance programmes, as well as LGBTQ + ‐relevant causes such as caring and fighting for patients living with HIV and partnering with trans patients to refine surgical GAC techniques [21, 22, 23, 24]. Advocacy is so central to the profession that the Accreditation Council for Graduate Medical Education requires that residents ‘must demonstrate competence in advocating for quality patient care and optimal patient care outcomes,’ as well as learning to ‘advocate for patients within the health care system to achieve the patient's and patient's family's care goals.’ [25]

The responsibility to advocate is guided by the four core tenants of bioethics: respect for autonomy, beneficence, nonmaleficence, and justice, all of which are threatened for trans patients by restrictions on gender‐affirming care. Not only does the restriction of care infringe on trans patients’ rights to bodily autonomy and gender self‐determination, but an environment in which physicians lack the resources, knowledge, or respect to discuss options for GAC also fundamentally limits the provision of true informed consent [26]. The well‐documented positive effects of GAC on mental health and social functioning require the physician to facilitate access to GAC when appropriate (beneficence) and oppose its undue restriction (non‐maleficence). The principle of justice requires that physicians must work to ensure that GAC is equitably accessible, including across such demographics as insurance status, income level, and geographical location, and is as accessible to trans patients as it is to cisgender patients when they seek the same medications or care.

Many providers lack the knowledge and formal training that they perceive necessary to do provide GAC or to be involved in advocacy [27]. Still others may, out of fear, apathy, or overwhelm, preemptively surrender to restrictions on GAC and consequent harm to trans patients. It is to these providers that we address the following recommendations and appeals for support from the trans community. Some providers may ‘conscientiously object’ to this care [28]. Strategies to reach this group of providers are unfortunately out of the scope of this manuscript.

Per respondents' recommendations, in the immediate and short term, providers must educate themselves and their colleagues on the experiences of their trans patients. Perhaps most pertinent for healthcare providers to understand are the concerns that trans participants report over access to both medical and surgical care. These concerns require nuanced and skilled shared decision‐making and require physicians to have a detailed and up‐to‐date knowledge of policies affecting trans care. They also require an understanding of historical and current alternative strategies for obtaining GAC [18] and decreased access to health insurance, making both harm reduction strategies and judicious use of ancillary services such as laboratory testing necessary for competent transgender care. Open, nonjudgmental discussion within the confines of provider‐patient confidentiality can be used to further elicit individual concerns and determine best options for medications and lab monitoring. Providers must also work to both acknowledge the very real mental health impacts of access‐restricting legislation and collaborate meaningfully with the trans community to combat potential consequences. These strategies must be informed by existing models of trans resilience, which utilise social support and social connectedness, hope, self‐advocacy, self‐definition of gender, gender affirmation, and identity pride [29, 30, 31]. Paired with the larger‐scale advocacy strategies delineated below, trans‐competent mental health interventions by providers from any specialty have the potential to impact the survival and quality of life of trans people in this environment.

In the medium term, it is more essential than ever that providers not only signal their LGBTQ+ allyship, but both visibly and substantively prioritise the physical and psychological safety of trans patients. Healthcare providers must be aware of the changing politics of gender expression that trans patients are being forced to navigate, and must provide a safe environment for patients of all gender identities and expressions. Allyship enactment can be as simple as introducing oneself with one's pronouns, or as strong as public statements, intentionally welcoming clinic design, and hiring or consulting trans community members about LGBTQ+ care. Importantly, allyship must transcend ‘virtue signalling’ and needs to include substantive resistance to threats made by political actors intended to suppress access to GAC. Notable examples of such resistance include appeals filed by several leading healthcare organisation in response to subpoenas by the Department of Justice [32].

In the longer term, providers must implement new strategies to minimise the impacts of access‐restricting legislation. Luckily, the trans community has provided a template for what these strategies could look like. Advocacy takes many forms. It can be an everyday act, like increasing psychological screening, implementing harm reduction strategies, and connecting patients to trans organisations or mutual aid. It can be introductions with pronouns, educating colleagues, or implementing sliding scale payment models. For some, advocacy for the trans community can be more public, like speaking to legislators, using legal pathways to resist legislation including writing amicus briefs and appealing federal attempts to interfere with care, and advocacy within and through professional organisations.

### 4.1 Call to Action

In this survey, the trans community reached out to the healthcare community for support. Clinically, it is essential for healthcare providers to understand the changing responses of the trans community to legislative shifts. Ethically, it is essential for healthcare providers to act. Our study lays out a clear framework for meaningful intervention, based on the needs of trans people, in a time of legislative and political persecution, described by one participant as the ‘current tilt towards trans eradication.’ Healthcare providers have a professional and ethical responsibility to advocate for patients who come to them imploring them to act. If healthcare providers fail to do so, they will irrevocably lose the trust and harm the long‐term health of the trans community. As one participant summed up their stance, ‘If I didn't have access to transition‐related care from medical professionals, I would leave the country. I would feel failed by the medical community entirely.’

### 4.2 Limitations

There are demographic categories that are over‐ or under‐represented in our sample. Our study participants were predominantly White and AFAB, which does not reflect the U.S. trans population as documented in other studies. Our sample is also not representative regarding educational status, with 77.0% holding a college degree or higher, whereas for the U.S. trans population as a whole, only 15% hold at least a college degree [33]. Similarly, only 23.6% of our respondents report a household income below $40,000, compared to 40% of U.S. trans adults [34]. This may have an impact on some responses—relocating geographically requires financial resources and employment flexibility and may be more within reach, and therefore more likely to be considered in our sample than in the U.S. trans population as a whole. As this sample was disproportionately White, had higher levels of educational attainment, and reported greater household income than the U.S. trans population, it must be recognised that trans people of colour or trans people with less formal education or household income may report different insights or experiences than what was captured by this survey.

Notably, the rapidly changing social and political landscape has continued to worsen for trans people in the US (and elsewhere) since this survey was administered, and many of the concerns raised by participants at the time of the survey have since become a reality, warranting ongoing attention to the changing needs and mental health of the trans community.

## 5 Conclusions

Trans attitudes and concerns around GAC are shifting with the changes occurring in the U.S. political environment. Survey respondents were almost universally concerned about trans people's access to gender‐affirming hormone therapy, and those interested were concerned about access to surgical interventions as well. Trans patients' reactions to these challenges are essential for healthcare providers to understand in order to provide high‐quality, equitable care to a population so heavily targeted by current legislation and policy.

## Author Contributions

**Kay Wallace:** writing – original draft, formal analysis, data curation, writing – review and editing, conceptualisation, investigation. **Jayne Reynolds:** investigation, methodology, writing – review and editing, validation, data curation, formal analysis. **Jesse Krikorian:** conceptualisation, writing – review and editing, writing – original draft, investigation. **Gaines Blasdel:** writing – review and editing, conceptualisation. **Mary Byrnes:** conceptualisation, methodology, validation, formal analysis. **Daphna Stroumsa:** conceptualisation, writing – review and editing, methodology, supervision, project administration.

## Ethics Statement

The study was reviewed by the University of Michigan IRB (HUM HUM00265503) and deemed exempt.

## Conflicts of Interest

The authors declare no conflicts of interest.

## Supporting information

Supporting File

## Data Availability

The data that support the findings of this study are available on request from the corresponding author (KW). The data are not publicly available by request of study participants.
