# Supplementary material for: ‘I Would Feel Failed By the Medical Community Entirely’: Transgender Americans' Calls for Advocacy From Health Professionals in a Care‐Restricted World
Source: Health Expect. 2026 Jun 12;29(3):e70716. doi: 10.1111/hex.70716 (PMC13261292; doi:10.1111/hex.70716)
Supplement: Supplementary file 1 — Supporting File [file HEX-29-e70716-s001.docx]

Researchers from the University of Michigan are conducting a study with Trans (transgender, nonbinary, genderqueer, and all other non-cisgender) people. We want to learn how you are thinking about potential changes to hormone access in the context of political shifts. Your thoughts will help guide clinics and doctors support our trans patients. Please complete this survey for a $50 mailed card or check.

Risks and discomforts: You may be asked personal questions regarding your gender, hormones, and health (including mental health) which may be difficult to answer. You can decline to answer any question.

Participating in this study is completely voluntary.

We will protect the confidentiality of your research records by storing your personal identifying information and recording of your interview in a secure database on a computer at the University of Michigan. We will remove all personal identifying information from the transcript that is made from the audio-recording of the interview.

At this point in this study, we are specifically looking for responses from people of color, thank you for understanding!

Are you over 18, currently living in the United States, a person of color, and do you identify as Trans, Transgender, nonbinary, GenderQueer, or other non-cisgender identity?

- Yes
- No

What is your name (chosen, or a name you would like us to use to refer to you)? ___________

How old are you? ____________

What is your gender identity? _____________

What is your race? Please mark all that apply

- Black or African-American
- White
- American Indian or Alaska Native
- Asian, Hawaiian or Pacific Islander
- Middle Eastern or Mediterranean
- Prefer to not disclose
- Other:

Are you of Hispanic, Latinx, or Spanish Origin?

- No, I am not Hispanic or Latinx
- Yes, I am Hispanic or Latinx

What best describes the area you live in?

- Urban
- Suburban
- Rural

Which region of the US do you live in?

- Northeast
- Midwest
- Pacific Northwest
- Southwest
- South
- Outside the continental US

Which State do you live in? ________________

What is your highest level of education?

- I didn't finish high school
- High school graduate
- Some college
- Professional degree
- 4 year or college degree
- Advanced degree

Are you currently employed?

- Unemployed
- Employed part time
- Employed full time
- Self employed
- Full time student
- Other:

What is your total household income?

- Less than $10,000
- 10,000 to 19,999
- 20,000 to 39,999
- 40,000 to 59,999
- 60,000 to 79,999
- 80,000 to 99,999
- 100,000 to 149,999
- 150,000 or more

How many people (NOT including yourself) are supported by that income? ___________

Do you have health insurance?

- No, I'm not insured
- Yes, Medicaid, Medicare, Indian Health Service or other public insurance
- Yes, through the VA
- Yes, private insurance through an employer
- Yes, private insurance through the marketplace
- Other, please describe:

Are you currently taking gender affirming hormones (HRT)?

- Yes, prescribed by a doctor, nurse, or other licensed professional
- Yes, I get them off the internet / from friends / other source
- Yes, both prescribed by a doctor, nurse, or other licensed professional AND off the internet / from friends / other source
- No

What was your sex assigned at birth?

- Male
- Female
- Intersex

How worried are you about your own ability to access hormones in the near future (within the next 4 years)?

- Very worried
- Somewhat worried
- Neutral
- Not that worried
- Not worried at all

What are you thinking about your own ability to access hormones in the near future?

Have your feelings about your own ability to get hormones changed following the recent elections?

- Yes
- No

How have your feelings changed? ___________________

How worried are you about other trans folks' ability to access hormones in the near future (within the next 4 years)?

- Very worried
- Somewhat worried
- Neutral
- Not that worried
- Not worried at all

What are you thinking about other trans folks' ability to access hormones in the near future? __________________

If your feelings about other trans folks' ability to get hormones have changed following the election, how have your feelings changed? __________________

Are you interested in gender affirming surgeries?

- Yes
- Unsure
- No - I don't think I want any surgeries
- No - I have completed all the surgeries I'm interested in for the time being

How worried are you about your own ability to access surgeries or other (non-hormone) gender affirming care in the near future (within the next 4 years)?

- Very worried
- Somewhat worried
- Neutral
- Not that worried
- Not worried at all

Which, if any, of the following are you doing in preparation for possible changes to hormone and care access? (mark all that apply)

- Stockpiling hormones
- Discussing this with friends
- Reaching out to my doctor for prescription changes
- Reaching out to my doctor for changes to my diagnoses, chart documentation, or other written documentation
- Researching ways to obtain hormones without a prescription (internet, friends)
- Researching ways to produce hormones at home
- Making changes to my legal documentation (sex and name change)
- Making plans to leave my state
- Have left my state
- Making plans to leave the country
- Have left the country
- Making plans to be stealth
- Making plans to de/re-transition
- Making plans to otherwise change my gender presentation
- None of these
- Other:

Based off your responses to the last question, please tell us what you are thinking about the following:

- Stockpiling hormones:
- Discussing this with friends:
- Reaching out to my doctor for prescription changes:
- Reaching out to my doctor for changes to my diagnoses, chart documentation, or other written documentation:
- Researching ways to obtain hormones without a prescription (internet, friends):
- Researching ways to produce hormones at home:
- Making changes to my legal documentation (sex and name change):
- Making plans to leave my state:
- Have left my state:
- Making plans to leave the country:
- Have left the country:
- Making plans to be stealth:
- Making plans to de/re-transition:
- Making plans to otherwise change my gender presentation:
- None of these:
- Other:

Is there anything else you want to share with us about this? ___________________

If accessing transition-related care becomes (more) difficult or not possible, what kind of support would be valuable to you - from healthcare professionals, community organizations, or other sources? ___________________

Do you currently have access to healthcare via telehealth (virtual, zoom, or phone) option?

- Yes
- No
- Not sure

Would telehealth (virtual, zoom, or phone) visits help make care more accessible to you?

- Yes
- No
- Not sure

Can we reach out to you to talk more about these issues in a (paid and confidential) interview?

- Yes
- No

The following questions are only for the purposes of contacting you to schedule the interview and send compensation for the interview.

- What is your email?
- What is your phone number?
- What is your full name and home address? We need this to send you your compensation for completing this survey. This information will be kept completely separate from your answers to the survey and interview. Thank you.

The topics covered in this survey can be distressing to consider. If you have concerns about your safety or are feeling suicidal, please reach out for help. Two options are dialing 988 for the national suicide and crisis hotline or reaching out to Trans Lifeline at (877) 565- 8860 between 1 PM – 9 PM Eastern Time. Of note, the Trans Lifeline is a trans-specific crisis hotline and will not call emergency services or law enforcement without your explicit request.
